# Depression, Anxiety, and Pain Predict Quality of Life in Patients with Differentiated Thyroid Cancer Postradiotherapy Ablation in Taiwan: A 48-Week Follow-Up Study

**DOI:** 10.3390/curroncol31110488

**Published:** 2024-10-24

**Authors:** Kuan-Ying Hsieh, Kai-Da Cheng, Che-Sheng Chu, Yung-Chieh Yen, Te-Chang Changchien

**Affiliations:** 1 Department of Child and Adolescent Psychiatry, Municipal Kai-Syuan Psychiatric Hospital, Kaohsiung 802211, Taiwan; isanrra@gmail.com; 2 Department of Physical Therapy, I-Shou University, Kaohsiung 840203, Taiwan; 3 Department of Community Psychiatry, Kaohsiung Municipal Kai-Syuan Psychiatric Hospital, Kaohsiung 802211, Taiwan; 4 Department of Nursing, Meiho University, Pingtung 906006, Taiwan; 5 Graduate Institute of Psychology, National Chung Cheng University, Chiayi 621301, Taiwan; 6 Department of Psychiatry, Kaohsiung Veterans General Hospital, Kaohsiung 813414, Taiwan; 7 Graduate Institute of Medicine, College of Medicine, Kaohsiung Medical University, Kaohsiung 807378, Taiwan; 8 Department of Psychiatry, E-Da Hospital, Kaohsiung 824005, Taiwan; 9 School of Medicine, College of Medicine, I-Shou University, Kaohsiung 840203, Taiwan

**Keywords:** depression, anxiety, pain, quality of life, differentiated thyroid cancer

## Abstract

Despite the generally good prognosis of differentiated thyroid cancer (DTC), impairments in health-related quality of life (HRQoL) remain a major concern in these patients. This study examined the patterns and predictors of change in mental and physical HRQoL in DTC survivors following radiotherapy ablation. Two hundred patients with DTC who received radiotherapy ablation in southern Taiwan between 2015 and 2018 were interviewed using the Taiwan version of the 36-item Short-form Health Survey (SF-36), the Taiwanese Depression Questionnaire (TDQ), and the Hamilton Rating Scale for Anxiety (HAM-A) at baseline and after 24 and 48 weeks of treatment. The demographic characteristics, thyroid-related factors, recombinant human thyroid-stimulating hormone (rhTSH), and serum calcium level were also collected. Improvements in both mental and physical HRQoL were noted over the first 24 weeks following radiotherapy ablation. Between 24 and 48 weeks, mental HRQoL was increasing, whereas physical HRQoL decreased. Higher levels of anxiety and pain predicted both poor physical and mental HRQoL. Further, higher levels of depression predicted poor mental HRQoL. Additionally, factors such as low income, rhTSH use, elevated TSH levels, low free T4, and higher physical HRQoL were associated with poorer mental HRQoL during the follow-up period. The early detection and intervention of depression, anxiety, and pain should be the objective for programs aiming to improve HRQoL.

## 1. Introduction

### 1.1. Differentiated Thyroid Cancer

The global incidence of thyroid cancer, the most common type of endocrine malignancy, has rapidly increased [1]. In Taiwan, it was the fourth most common cancer among women in 2016 with an incidence of 18.1 per 100,000 [2]. Differentiated thyroid cancer (DTC), including the papillary and follicular types, accounts for more than 90% of all thyroid cancers [3]. Patients with DTC generally show good prognosis [4], and survival rates are improving. Standard treatments include surgery, radioactive iodine-131 (RAI), and thyroid hormone suppression therapy [5]. The long-term physical and psychological issues experienced by DTC survivors warrant further attention and investigation.

### 1.2. Mental Health in Thyroid Cancer Patients

Although the prognosis for thyroid cancer is better than for other cancers, patients often experience psychological distress upon diagnosis [6,7], which may vary over the course of cancer treatment. Reactions to diagnosis are wide ranging [8]. The treatment of thyroid cancer may cause a feeling of weakness in the patient and inability to follow treatment regimens. Symptoms can significantly impact social roles, personal life, and appearance. Furthermore, patients receiving RAI must be housed in radiation isolation wards, resulting in fear of radiation exposure, loneliness, and pessimism, which could negatively affect mental health. The precise psychological impacts varied across different studies. Increased anxiety and/or depression in thyroid cancer patients has been reported [9,10]. Associations of sex and age with anxiety and depression were observed in DTC patients [11,12]. Therefore, mental health concerns in thyroid cancer patients require particular attention, especially given their interrelationship with physical health.

### 1.3. Quality of Life in Thyroid Cancer Patients

Health-related quality of life (HRQoL) reflects individuals’ subjective perceptions of their capacity to perform significant activities influenced by their health status [13], encompassing both positive and negative aspects of physical, mental, and social well-being. Cancer treatment aims not only to improve survival rates but also to maintain HRQoL [14]. However, many cancer survivors face challenges in adapting to life post-treatment, highlighting the importance of assessing HRQoL in their ongoing care [15]. 

HRQoL can reflect the therapeutic effects of any intervention more accurately than survival or fatality rates [7]. Previous studies involving HRQoL in patients with DTC have shown conflicting findings. Research indicates that thyroid cancer patients experience impaired health-related quality of life (HRQoL) compared to the general population [16,17]. One study investigating 75 subjects with DTC showed that 82.7% considered their health to be the same as or better than baseline. Patients diagnosed with DTC at more than 45 years old reported significantly less pain than those diagnosed earlier [18]. Other studies found that patients with thyroid cancer had impaired HRQoL [9,19,20]. Despite better prognosis, the QoL for DTC patients is below that of the general population and other cancers [21,22]. Further, emotional distress was a major determinant of reduced QoL in patients with DTC [10].

### 1.4. Indicators of HRQoL in Patients with DTC

Several factors influence the QoL of patients with thyroid cancer with both physical and psychological impacts. Positive indicators of HRQoL include male aged younger than 45 years, with partner, radioiodine therapy [23], surgery quality [24,25], use of recombinant human thyroid-stimulating hormone (rhTSH) [26], psychological and behavioral interventions [27], and relationship with physician [28]. Negative HRQoL indicators in patients with DTC are older age, advanced staging at diagnosis [29], and hormonal withdrawal [26]. 

These psychological issues, along with self-esteem, are strongly correlated with HRQoL, with depression being the most influential factor in thyroid cancer patients [30]. Risk factors for anxiety, depression, and reduced HRQoL include socioeconomic factors, disease-specific factors, management factors, comorbidities, and patient perceptions [17]. Protective factors against depression and anxiety include social support and a strong sense of coherence [31]. Notably, thyroid cancer survivors experience poorer HRQoL compared not only to the general population but also to those with benign thyroid pathology and survivors of other cancer types [17]. These findings highlight the importance of addressing psychological well-being in thyroid cancer patient care. Furthermore, pain is associated with psychological distress and physical HRQoL. However, there is a paucity of studies that have demonstrated a relationship between pain and HRQoL in patients with DTC.

### 1.5. Aims

This study examined predictive effects of disease-specific predictors, anxiety, depression, and pain for HRQoL for DTC survivors following radiotherapy ablation. We hypothesized that depression, anxiety, and pain negatively predict HRQoL in DTC survivors.

## 2. Materials and Methods

### 2.1. Participants and Ethics

Participants were recruited between January 2015 and December 2018 from the E-Da Hospital in Taiwan. Inclusion criteria were DTC diagnosis, aged between 20 and 85 years, post-thyroid surgery and radiotherapy ablation treatment, and free of cancer metastasis. Written informed consent was obtained from all participants before assessment in a face-to-face interview. We excluded those who showed any cognitive deficits that could have prevented them from understanding the study purpose or completing the questionnaire.

The study was approved by the institutional review board of E-Da Hospital (protocol number: EMRP01102N), which was conditional on obtaining informed consent from all study participants.

### 2.2. Measures

#### 2.2.1. Taiwanese Depression Questionnaire

We used the Taiwanese Depression Questionnaire (TDQ) to evaluate the level of depressive symptoms. The TDQ is a culturally relevant tool developed for screening depression in Taiwanese populations [32]. It has demonstrated high validity and reliability in various contexts, including cancer patients and those with chronic pain. In a study of head and neck cancer patients, the TDQ showed comparable validity to the Hospital Anxiety and Depression Scale for depression screening [33]. When compared to the Beck Depression Inventory for screening depression in chronic pain patients, the TDQ showed a trend of better validity, particularly in its cognitive/affective components [34]. These findings suggest that the TDQ is an effective tool for detecting depression in various Taiwanese patient populations, including those with cancer and chronic pain.

The 18 items of the TDQ are assessed on a scale from 0 to 3 points. The subjects rate each item in terms of certain physical and emotional feelings during the previous week. TDQ scores range from 0 to 54. Higher total scores indicate more severe depressive symptoms. The internal reliabilities of the TDQ for this study, measured using Cronbach’s α, were 0.88 (baseline), 0.84 (24 weeks), and 0.89 (48 weeks).

#### 2.2.2. Hamilton Rating Scale for Anxiety

The Hamilton Rating Scale for Anxiety (HAM-A) is a clinician-rated questionnaire used to access the severity of anxiety symptoms [35]. Research has shown that anxiety is prevalent among cancer patients, particularly those with terminal diagnoses, and can significantly impact quality of life [36]. A pilot randomized controlled trial demonstrated that brief, tailored CBT was feasible and effective in reducing anxiety symptoms in patients with terminal cancer, as measured by the Hamilton Anxiety Rating Scale (HAM-A) [36].

This commonly used scale incorporates 14 items, which are each defined by a series of symptoms, measuring psychic (mental agitation and psychological distress) and somatic (physical complaints that are related to anxiety) anxiety. Each of the 14 items is scored on a scale from 0 (absent) to 4 (severe), producing a total score range of 0–56. Higher total scores indicate more severe anxiety symptoms. The internal reliabilities of the HAM-A for this study, assessed using Cronbach’s α, were 0.73 (baseline), 0.69 (24 weeks), and 0.67 (48 weeks).

#### 2.2.3. Thirty-Six Item Short-Form Health Survey

The self-administered 36-Item Short-form Health Survey (SF-36) was adopted to assess HRQoL in the month preceding the administration of the survey. Its 1996 translation into Chinese for use in the Taiwanese population has shown good psychometric properties [37]. The Medical Outcomes Study of SF-36 has been validated for assessing quality of life in various cancer populations. In lung cancer patients, the SF-36 demonstrated poorer quality of life across all domains compared to healthy controls with physical aspects being particularly affected [38]. For breast cancer patients, the Chinese version of SF-36v2 showed good reliability, validity, and sensitivity [39]. In brain tumor patients, the SF-36 exhibited adequate internal consistency for most subscales and demonstrated good construct validity, correlating well with measures of depression and functional status [40]. Overall, these studies support the SF-36 as a reliable and valid instrument for assessing health-related quality of life in cancer patients across different types of malignancies.

The SF-36 measures eight domains of HRQoL, including physical functioning, role limitations due to physical health problems, bodily pain (BP), general health perceptions, vitality, social functioning, role limitations, and mental health. This instrument allows two summary scores to be calculated for HRQoL: the physical component summary (PCS) and the mental component summary (MCS). For each domain, a score was calculated and was transformed to a value from 0 to 100 [41]. Relatively high total scores indicate better HRQoL. The internal reliabilities of the SF-36 for this study, assessed using Cronbach’s α, were 0.90 (baseline), 0.87 (24 weeks), and 0.90 (48 weeks).

We also used the BP domain of HRQoL to evaluate pain. Higher total scores indicate lower BP. The internal reliabilities of the BP domain of HRQoL for this study, assessed using Cronbach’s α, were 0.81 (baseline), 0.82 (24 weeks), and 0.83 (48 weeks).

### 2.3. Study Procedures

A total of 200 patients with DTC agreed to participate in this 48-week follow-up study and underwent intake interviews to provide baseline data before beginning therapeutic RAI treatment.

They completed questionnaires, including the provision of sociodemographic data, their clinical history (including previous psychiatric services, rhTSH use, and thyroid function levels), and completion of symptom-rating instruments for depression and anxiety and their HRQoL at the intake interview. At each follow-up interview, thyroid function, levels of depression and anxiety, and HRQoL were reassessed.

### 2.4. Statistical Analysis

We employed an intention-to-treat (ITT) analysis in this study. Data analyses were performed using the software Statistical Package for the Social Sciences 17.0 (SPSS, Chicago, IL, USA). The baseline characteristics of the participants were analyzed using descriptive statistics. The correlations among the levels of TDQ, HAM-A, SF-36-BP, SF-36-MCS, and SF-36-PCS at each follow-up interview were examined using Pearson’s correlation.

We used the generalized estimating equation (GEE) with a first-order autoregressive working correlation structure [42] to determine the independent predictors of HRQoL. The scores for the QoL domains of MCS and PCS were used as dependent variables at the follow-up interviews. The factors collected at intake (demographic characteristics, previous psychiatric service, free T4, TSH, thyroglobulin, antithyroglobulin antibodies, rhTSH use, calcium, and the scores for TDQ, HAM-A and SF-36 at intake) and those collected at follow-up (TDQ, HAM-A, and SF36) were the independent variables. Changes in HRQoL between intake and follow-up points were also analyzed using GEE. The correlation models were autoregressive. We drew inferences at the 0.05 significance level for all inferential statistical procedures.

## 3. Results

### 3.1. Subjects

The 200 participants’ sociodemographic characteristics, thyroid function, calcium level, and rhTSH use are shown in Table 1. Out of 200 participants, 114 completed the 48-week follow-up, while 86 (43%) withdrew during the course of the study. Participants who discontinued the study were classified as ‘dropouts’, while those who completed the study were designated as ‘completers’. A comparison between the completers and dropouts is presented in Table 1. 

The majority of participants were female, comprising over 70% of the sample. There were no statistically significant differences between completers and dropouts in terms of gender, age, education, partnership status, income, history of psychiatric services, baseline free T4, baseline thyroglobulin, baseline antithyroglobulin antibodies, or rhTSH use, with the exception of baseline TSH levels.

### 3.2. Improvement of Depression, Anxiety, Pain, and HRQoL

**Figure 1:**
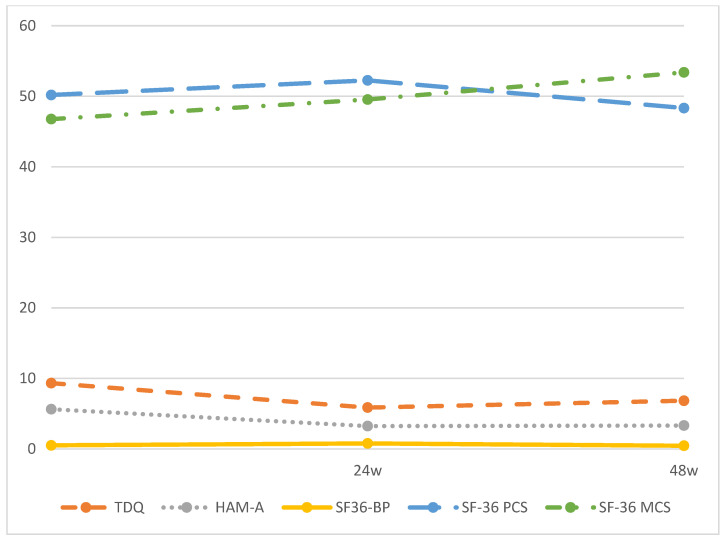
TDQ, HAM-A, SF-36 BP, SF-36 PCS and SF-36 MCS scores during the 48-weeks follow-up period. Note: TDQ, Taiwanese depression questionnaire; HAM-A, Hamilton anxiety scale; SF-36 BP, Short Form-36 Health Survey, Bodily Pain; SF-36 MCS, Short Form-36 Health Survey, Mental Component Summary; SF-36 PCS, Short Form-36 Health Survey, Physical Component Summary.

In all, 149 (74.5%) and 114 (57%) participants attended follow-up interviews at 24 and 48 weeks after intake, respectively. Changes in depression, anxiety, pain, and HRQoL levels during the 48-week period after ablation are shown in Figure 1 and Table 2. Improvements in mental and physical HRQoL were noted in the first 24 weeks following ablation. During the period from 24 to 48 weeks, mental HRQoL continued to increase, while physical HRQoL decreased. We found that the scores for all QoL domains at each follow-up interview differed significantly from those at the intake interview (*p* < 0.01). 

Our post hoc analysis revealed significant differences in TDQ and SF-36 MCS scores across the three follow-up time points. However, HAM-A scores showed significant differences only between baseline and 24 weeks, and between baseline and 48 weeks, with no significant difference between 24 weeks and 48 weeks. Similarly, SF-36 BP and SF-36 PCS scores followed the same pattern with significant differences observed between baseline and 24 weeks, and between 24 weeks and 48 weeks, but not between baseline and 48 weeks. These results suggest that while pain and physical health returned to baseline levels in patients with DTC over the one-year follow-up period, their depression, anxiety, and mental health demonstrated improvements compared to baseline.

The scores for TDQ, HAM-A, and SF-36 BP at each follow-up interview and their cross-sectional correlations with the levels of HRQoL at each follow-up interview are shown in Table 3, respectively. The cross-sectional correlations among TDQ, HAM-A, and the levels of HRQoL at each follow-up interview were all significant (*p* < 0.01, with the exception of SF-36 BP).

The results of the GEE for the analyses of the predictors of mental and physical HRQoL during the 48-month period are shown in Table 4. Lower levels of anxiety (HAM-A, β = −1.12, *p* < 0.001; β = −0.45, *p* < 0.01) and lower levels of pain (SF-36 BP, β = 2.74, *p* < 0.05; β = 5.66, *p* < 0.001) predicted higher levels of mental and physical HRQoL. Low income (β = −4.74, *p* < 0.05), rhTSH use (β = −5.67, *p* < 0.05), high TSH level (β = −0.02, *p* < 0.001), low free T4 level (β = 2.55, *p* < 0.05), high depression level (β = −0.37, *p* < 0.05), and high physical HRQoL level (β = −0.45, *p* < 0.001) predicted poor mental HRQoL. Meanwhile, not having had psychiatric services (β = 4.07, *p* < 0.001) and high mental HRQoL (β = −0.23, *p* < 0.001) predicted poor physical HRQoL.

## 4. Discussion

### 4.1. Main Findings of This Study

We found that anxiety and pain were negative predictors for both physical and mental HRQoL in DTC survivors. Low income, rhTSH use, high TSH, low free T4, high depression, and high physical HRQoL showed poor mental HRQoL during follow-up. Patients who had no psychiatric service before DTC treatment and high mental HRQoL had poor physical HRQoL. There are few longitudinal studies that have focused on changes in HRQoL in patients with DTC, specifically in relation to mental health. To the best of our knowledge, our longitudinal study is one of the few to explore the possible influence of mental health and the relationship between mental health and HRQoL, presenting certain notable findings after adjustment for common demographic and clinical data.

### 4.2. Thyroid Cancer and Mental HRQoL

Psychological distress, including increased anxiety and/or depression symptoms, is important for DTC patients. However, disease-specific predictors, such as TSH, were not previously identified. We found that patients with higher TSH levels and lower free T4 levels had lower mental HRQoL at a 48-week follow-up.

Thyroid autoimmunity was associated with depression and bipolar disorder [43]. However, the mechanism for this remains unclear. One study suggested that thyroid antibodies bind directly to the central nervous system [44]. Furthermore, dysfunction of the blood–cerebrospinal fluid barrier is determined to be owing to the specific binding of antithyroglobulin antibodies to cerebellar vascular smooth muscle cells [45]. A cross-sectional study conducted in Germany reported that elevated antithyroglobulin titer was a predictor for depression in DTC patients [46]. However, we did not identify an association between antithyroglobulin antibodies and HRQoL in the mental or physical domain.

### 4.3. Thyroid Cancer and Physical HRQoL

Thyroid cancer treatment has three main steps: surgery, radioiodine ablation, and hormone treatment with TSH suppression. At 4–6 weeks after surgery, patients must be checked to ensure the absence of any thyroid hormone supplement. Symptoms of hypothyroidism, including fatigue, weight gain, cramps, irritability, and memory impairment, could produce low HRQoL [28]. Another study of 150 DTC patients following thyroid hormone withdrawal identified correlators of HRQoL [47]. Most of the important factors were physical, including fatigue, intolerance of cold or heat, sleep disturbance, and weight gain. A cross-sectional study found that postoperative radioiodine ablation was the most important predictor for physical HRQoL in patients with thyroid cancer [48]. Therefore, it is necessary for HRQoL to identify physical symptoms during the course of thyroid cancer treatment.

In our study, only 11% of participants had previously received psychiatric services. This group had better physical HRQoL than the others. Psychiatrists routinely assess thyroid function and perform physical examinations as part of standard clinical practice. Research indicates that thyroid screening during psychiatric evaluations and other medical assessments can facilitate the early detection of thyroid disorders and malignancies. For instance, Radhakrishnan et al. (2013) reported a high prevalence of thyroid dysfunction among psychiatric inpatients [49]. This suggests that patients with differentiated thyroid cancer (DTC) who regularly consult psychiatrists may benefit from earlier detection compared to those who do not engage in psychiatric care.

### 4.4. Pain and HRQoL

Neck dissection influences HRQoL in both positive and negative ways in DTC patients [24]. Positively, it can lead to appropriate surgery with a good outcome, including the removal of pathological tissue. Negative effects include postoperative complications of anesthesia, numbness, neuropathic pain, edema and limitations to the neck/shoulder movement, and reduced speech and eating abilities, all of which influence HRQoL. Among patients receiving neck dissection, 70% reported shoulder pain [50]. Other studies found that patients who have undergone surgery for thyroid cancer have worse mental and physical HRQoL scores than the general population [48,51]. Is mental or physical HRQoL recoverable over time? Two prospective studies addressed this. One found a trend toward recovery 12 months after operation [52]. The second, a prospective study, demonstrated that postoperative HRQoL scores, especially in eating and emotional function, recover in 12 months after surgery [53]. Pain is therefore important for predicting HRQoL in patients with thyroid cancer after surgery.

Pain is associated with psychological distress [54]. It has similar neurophysiological mechanisms to depression and anxiety, such as the dysregulation of the anterior cingulate cortex, the insular cortex, and the ventral tegmental area [55]. The glutamate signal pathway has a major role in regulating pain and emotion [56]. In examining pain in patients with DTC, comorbidity with depression and anxiety should also be taken into consideration.

We found that pain is an independent predictor of HRQoL even after anxiety and depression in the 48-week follow-up period are controlled. For this reason, pain is not only the cause of poor mental or physical HRQoL but could also serve as an HRQoL detector at follow-up. Outpatient clinics in Taiwan are very busy, and doctors must often evaluate a patient in less than 10 min. Therefore, concise but important questions should be posed. Anxiety and depression are important predictors in our study, but the evaluation of mental status is not easy to perform in 10 min, especially by endocrinologists without psychiatric training. Taiwanese tend to avoid receiving psychiatric services due to stigma. We propose that endocrinologists use pain as a detector of HRQoL in patients with DTC following radiotherapy ablation and potentially refer those with profound pain to psychiatric services.

### 4.5. Thyroid Hormone Supplementation and HRQoL

It is reasonable to believe that thyroid hormone supplementation could improve HRQoL in patients with thyroid cancer. A randomized blind placebo control study compared QoL in patients receiving rhTSH with that of those who did not, and the rhTSH group showed better HRQoL and mood than the control group [26]. Two randomized controlled trials were conducted focusing on radiotherapy ablation’s effects on HRQoL with or without rhTSH [20,57]. In both, the HRQoL of euthyroid patients receiving rhTSH was preserved relative to a drop in HRQoL for hypothyroid patients not receiving rhTSH. Another prospective study found that patients had better SF-36 PCS scores after rhTSH treatment than the general US population but worse SF-36 MCS [58]. Thyroid hormone withdrawal (THW) caused transient but clinically significant deterioration in well-being particularly in younger patients [59]. Those study focused on physical domains of quality of life, mostly. The asynchronous results for the PCS and MCS are worth attention.

We found that patients receiving rhTSH exhibited poorer mental HRQoL compared to others, even after adjusting for thyroid function, while no significant effect was observed on physical HRQoL. Interestingly, previous studies have shown that rhTSH can help maintain quality of life scores at or above population norms in thyroid cancer patients [58,59]. In our univariate regression analysis, no significant predictors for rhTSH use were identified, including prior psychiatric history and baseline thyroid function, except for age (β = 0.04, *p* = 0.035). However, this association with age was not upheld in the multiple regression analysis. These findings warrant further investigation.

### 4.6. Parathyroid Hormone and HRQoL

Permanent hypoparathyroidism (PH) was defined as having parathyroid hormone levels going below the reference value at the time point of postoperative counseling and showing hypocalcemia continuing more than 12 months post-thyroidectomy [46]. In our study, we used serum calcium as an indicator for PH. A previous study investigating 2584 patients following thyroid cancer surgery found that low calcium was associated with low energy and fatigue [60]. Another multicenter study identified negatively impacted QoL in thyroid cancer PH [61]. In post-thyroidectomy patients, those with PH showed lower HRQoL than those without PH or with healthy controls [62]. PH has been found to be a predictor for anxiety in DTC patients [46]. Our finding for PH was not a predictor for HRQoL, which could be related to the effects of anxiety.

Finally, we found that physical HRQoL negatively predicted mental HRQoL and vice versa. Both mental and physical HRQoL showed improvements over baseline within 24 weeks. However, mental HRQoL increased during the 24th to the 48th weeks, while physical HRQoL decreased. The psychological distress of cancer could be resolved over time, but physical conditions (side effects, recurrence) may be emerging. Conversely, patients who have poor mental health may have lower levels of interest and social withdrawal, which reduce their needs and their awareness of physical condition.

This study had several limitations. First, our data were self-reported, and shared-method variance may have been a factor. Second, the single-center nature of this study may limit its generalizability with potential selection bias. Third, we did not evaluate the physical symptoms of hypothyroidism in this study. Fourth, we did not ascertain any comorbidities in this study. Fifth, the impact of patient dropouts on study outcomes is lacking, although we employed an ITT approach in this study. The ITT approach entails including all participants as originally allocated to their respective groups regardless of their adherence to the intervention or completion of the study protocol. This method preserves the benefits of randomization and provides a conservative estimate of treatment effects, closely reflecting real-world clinical outcomes. Sixth, the thyroid function test was not performed during the follow-up period, thus precluding the observation of any subsequent alterations in thyroid function. 

## 5. Conclusions

In conclusion, we identified that low income, rhTSH use, TSH, free T4, depression, anxiety, and pain were predictors for mental HRQoL in DTC survivors. Having received psychiatric services, anxiety, and pain were predictors for physical HRQoL in DTC patients. Further studies with extended follow-up periods, including ongoing monitoring of thyroid function, would be valuable in assessing the long-term effects of rhTSH on mental HRQoL. The physicians that were involved in the follow-up of DTC patients should devote particular attention to depression, anxiety, and pain in DTC patients following radiotherapy ablation.

## Figures and Tables

**Table 1:** Comparison of baseline demographic characteristics in completers (n = 114) and dropouts (n = 86).

	Completers (n = 114)	Dropouts (n = 86)	
	n (%) or Mean (SD)	n (%) or Mean (SD)	*p*
Female	85 (74.6)	63 (73.3)	0.84
Age (years)	50.8 ± 12.7	47.5 ± 12.6	0.07
Education level (years)	11.2 ± 4.3	12.0 ± 3.9	0.17
With partner	84 (74.3)	62 (72.1)	0.72
Low income	54 (47.8)	37 (43.5)	0.55
Previous psychiatric service	13 (11.4)	9 (10.5)	0.83
Baseline free T4 (ng/dL)	0.4 ± 0.8	0.6 ± 0.9	0.23
Baseline TSH (μIU/mL)	75.0 ± 38.9	104.6 ± 88.0	0.002 **
Baseline thyroglobulin (ng/mL)	394.2 ± 2121.4	111.2 ± 552.1	0.25
Baseline antithyroglobulin antibodies	17 (15.9)	8 (10.8)	0.33
rhTSH use	11 (9.7)	9 (10.6)	0.84

Note: SD, standard deviation; TSH, thyroid-stimulating hormone; free T4, free thyroxine; rhTSH, recombinant human thyroid-stimulating hormone. **, *p* < 0.01.

**Table 2:** Levels of TDQ, HAM-A, SF36 BP, SF36 MCS, and SF36 PCS at follow-up interviews.

	Baseline (n = 200)	24 Weeks (n = 149)	48 Weeks (n = 114)
TDQ, mean ± SD	9.3 ± 8.2	5.9 ± 4.8	6.8 ± 6.7
HAM-A, mean ± SD	5.6 ± 4.0	3.3 ± 3.1	3.3 ± 2.8
SF-36 BP, mean ± SD	0.5 ± 0.9	0.7 ± 0.7	0.5 ± 1.3
SF-36 MCS, mean ± SD	46.8 ± 10.4	49.5 ± 8.3	53.4 ± 12.4
SF-36 PCS, mean ± SD	50.2 ± 7.8	52.3 ± 7.6	48.3 ± 10.1

Note: SD, standard deviation; TDQ, Taiwanese Depression Questionnaire; HAM-A, Hamilton Anxiety Scale; SF-36 BP, Short Form-36 Health Survey, Bodily Pain; SF-36 MCS, Short Form-36 Health Survey, Mental Component Summary; SF-36 PCS, Short Form-36 Health Survey, Physical Component Summary.

**Table 3:** Pearson correlation coefficient matrix among levels of TDQ, HAM-A, and SF-36.

	Baseline			24 Weeks			48 Weeks		
	TDQ	HAM-A	SF-36 BP	TDQ	HAM-A	SF-36 BP	TDQ	HAM-A	SF-36 BP
SF-36 MCS	−0.53 **	−0.046 **	0.15	−0.50 **	−0.40 **	0.15	−0.044 **	−0.35 **	0.21 *
SF-36 PCS	−0.36 **	−0.31 **	0.09	−0.47 **	−0.26 **	0.17	−0.34 **	−0.26 **	0.79 **

Note: TDQ, Taiwanese Depression Questionnaire; HAM-A, Hamilton Anxiety Scale; SF-36 BP, Short Form-36 Health Survey, Bodily Pain; SF-36 MCS, Short Form-36 Health Survey, Mental Component Summary; SF-36 PCS, Short Form-36 Health Survey, Physical Component Summary. *, *p* < 0.05; **, *p* < 0.01.

**Table 4:** Predictors of HRQoL in patients with thyroid cancer receiving radioactive iodine treatment.

	SF36 MCS			SF36 PCS		
	*β*	95% CI		*β*	95% CI	
		Lower	Upper		Lower	Upper
Gender (ref: male)	2.64	−0.80	6.08	0.32	−2.42	3.05
Age	0.03	−0.18	0.24	−0.02	−0.13	0.10
Education level	−0.19	−0.71	0.33	0.05	−0.31	0.41
With partner	0.89	−2.66	4.44	0.29	−2.33	2.90
Low income	−4.74 *	−8.50	−0.99	−2.11	−4.75	0.52
Previous psychiatric service	2.74	−2.51	7.99	4.07 ***	1.77	6.37
rhTSH use	−5.67 *	−11.01	−0.32	−1.36	−7.11	4.40
TSH	−0.02 ***	−0.03	−0.01	0.00	−0.02	0.01
Free_T4	2.55 *	0.48	4.63	0.64	−1.44	2.72
Thyroglobulin	<−0.001	<−0.001	<0.001	<−0.001	<−0.001	<0.001
Anti-thyroglobulin antibodies	−2.43	−6.73	1.87	1.52	−1.11	4.16
Ca	1.97	−0.46	4.39	0.53	−1.06	2.12
TDQ	−0.37 *	−0.67	−0.07	−0.09	−0.26	0.07
HAM-A	−1.12 ***	−1.65	−0.59	−0.45 **	−0.78	−0.12
SF-36 BP	2.74 *	0.26	5.22	5.66 ***	4.48	6.83
SF-36 MCS				−0.23 ***	−0.34	−0.12
SF-36 PCS	−0.45 ***	−0.67	−0.24			

Note: CI, confidence interval; Ref, reference; TDQ, Taiwanese Depression Questionnaire; HAM-A, Hamilton Anxiety Scale; SF-36 BP, Short Form-36 Health Survey, Bodily Pain; SF-36 MCS, Short Form-36 Health Survey, Mental Component Summary; SF-36 PCS, Short Form-36 Health Survey, Physical Component Summary; TSH, thyroid-stimulating hormone; free T4, free thyroxine; rhTSH, recombinant human thyroid-stimulating hormone. *, *p* < 0.05; **, *p* < 0.01 ***, *p* < 0.001.

## Data Availability

The original contributions presented in the study are included in the article; further inquiries can be directed to the corresponding authors.
